# Mechanical Analysis and Verification Research on Asymmetric Four-Point Bending for the JCO Forming Process of LSAW Pipes

**DOI:** 10.3390/ma19050914

**Published:** 2026-02-27

**Authors:** Zhiyuan Zhang, Yi Liu, Zhiwen Lu, Junfang Shen, Yan Gao, Yize Chen

**Affiliations:** 1 School of Intelligent Manufacturing, Luoyang Institute of Science and Technology, Luoyang 471023, China; zhiyuan_zhang@lit.edu.cn (Z.Z.); lzw@lit.edu.cn (Z.L.); shen@lit.edu.cn (J.S.); 2 He’nan Institute of Metallurgy Co., Ltd., Zhengzhou 450000, China; gaoyan@hnas.ac.cn; 3 Anyang Iron and Steel Group Co., Ltd., Anyang 455004, China; gaoxiaowenan@163.com

**Keywords:** LSAW pipe, JCO forming process, four-point air bending, asymmetric air bending

## Abstract

Large-diameter longitudinal submerged arc welded (LSAW) pipes represent a critical component of long-distance oil and gas transmission pipelines. To enhance the forming efficiency of the JCO (J-shape to C-shape to O-shape) forming process for LSAW pipes, and to reduce residual straight segment in order to minimize the ovality of the formed pipes, an asymmetric four-point air bending (AFB) process was proposed. In this process, one end of the sheet contacts the dies with a straight segment, while the other end contacts a circular arc segment. The distribution of bending moments and mechanical model under different bending stages were analyzed, and analytical formulas for the main forming indexes before and after springback were derived. Experimental and finite element simulation verification were conducted for the AFB process. The results indicated that the error between the experimental and simulation results and the theoretical results was small, and the variation trends were consistent. Furthermore, the ellipticity of the pipes formed by the AFB process was less than 0.66%, which is obviously lower than that of the pipe formed by the symmetric four-point air bending (SFB) process. The forming quality and production efficiency of the pipe is improved, thereby proving the feasibility and reliability of the AFB process and promoting the development of LSAW pipe JCO forming processes.

## 1. Introduction

Large-scale longitudinally submerged arc welded (LSAW) pipes, which possess the advantages of short unit weld length and uniform weld and stress distribution, are primarily utilized in the construction of transportation pipelines in complex environments such as earthquake-prone regions, permafrost areas, seabeds, and regions with significant elevation differences. These pipes facilitate the long-distance transportation of large volumes of natural gas, crude oil, refined oil products, and other commodities. With the development of the global economy, the requirements for oil and gas storage and transportation pipeline construction and pipeline quality have increased to meet the global demand for oil and gas transportation in recent years [1,2,3].

**Figure 1:**
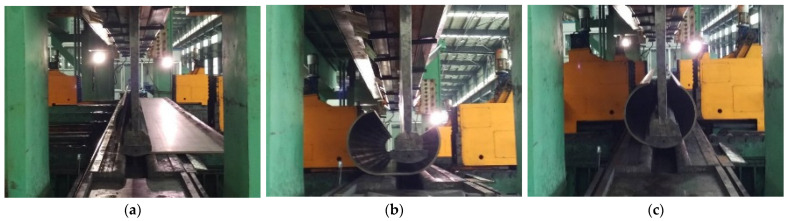
JCO forming process of LSAW pipe. (**a**) ‘J’ forming; (**b**) ‘C’ forming; (**c**) ‘O’ forming.

Due to its low investment requirements and wide production specification range, the JCO forming process for LSAW pipes, despite having relatively low forming efficiency, has been widely adopted as a manufacturing process for high-quality LSAW pipes, particularly in developing countries with limited financial resources [4,5]. Currently, most manufacturers continue to employ the traditional three-point JCO forming process for LSAW pipes, as illustrated in Figure 1. This process involves bending one side of the steel sheet with initially crimped edges into a ‘J’ shape through multiple concentric bending passes. The other side is then bent in the same manner, forming a ‘C’ shaped sheet. Subsequently, the sheet is joined to create an open-seam ‘O’ shaped pipe.

**Figure 2:**
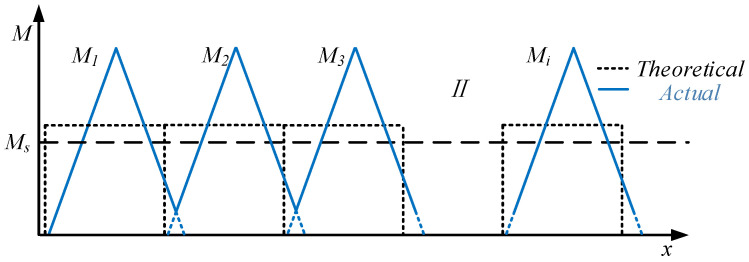
Bending moment distribution of JCO forming with three-point air bending process.

The bending moment distribution during the forming process is illustrated in Figure 2 [6], which demonstrates that the process is bending the sheet for multiple steps to incrementally form a pipe. Where $M_{s}$ is the elastic ultimate bending moment, $M_{1}$, $M_{2}$ $M_{3}$, $M_{i}$ represent the forming bending moments of steps 1, 2, 3, and i respectively, and x represents the length of the sheet. The uneven distribution of the bending moment results in a long residual straight segment.

To enhance the quality of LSAW pipes, numerous researchers have investigated the three-point bending JCO forming process. Y.Z. [7] examined the principle of three-point bending incremental forming. Y.Y. [8] derived the theoretical prediction model for residual strain and residual deformation in high-grade pipeline steel during bending. L.Q. et al. [9] studied and analyzed the influence of die curvature radius on forming results, while W.M. et al. [10] investigated the impact of single pass width in the forming process. Additionally, Y.Y. [11] analyzed the effect of punch reduction in the forming process. However, these researchers only considered the influence of a single parameter, failing to fully account for other key parameters, thereby limiting the persuasiveness of their findings. F.L. and Z.J. et al. [12,13] analyzed the asymmetric three-point bending process of sheets, concluding that springback is directly related to the residual stress level, with higher residual stress levels observed closer to the geometric neutral layer. Nevertheless, their analysis was limited to the asymmetric forming process of a single pass, which could not effectively explain the overall forming results of the entire process. Z.Y. [14] conducted theoretical research and model construction on the JCO incremental bending process. G.Y. and W.G. et al. [15,16] studied the forming process through numerical simulation, comprehensively analyzing the influence of various process parameters on forming outcomes. Building upon the theoretical analysis of the JCO three-point bending incremental forming process, Q.X. et al. [17,18,19] established a JCOE forming computer-aided process planning(CAPP) system for LSAW pipes capable of formulating process parameters. Z.Y. [20] researched an intelligent control system for JCO bending process. Despite the comprehensive studies by the aforementioned researchers on the JCO three-point bending incremental forming process, the inherent defect of the process remains: the uneven distribution of bending moments, resulting in many unformed parts (long residual straight segments) and significant residual stresses, causing the formed pipes to have large ellipticity and poor dimensional quality.

Practice has demonstrated that ellipticity increases with the extension of the residual straight segment length, which significantly impacts the quality of formed pipes. However, this issue has been scarcely reported in the existing literature. The current pipeline standard, API (American Petroleum Institute) Spec 5L, specifies that pipe ellipticity shall not exceed 1.5% of the diameter (0.75% for offshore applications).

**Figure 3:**
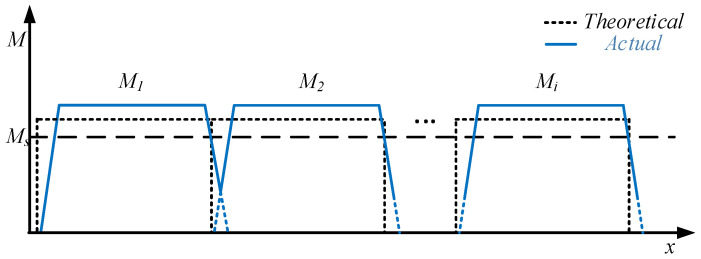
Bending moment distribution of JCO forming with the SFB process.

In view of this, S.H. et al. [21,22,23] proposed a symmetrical four-point bending (SFB) JCOC forming process for LSAW pipe, replacing the traditional three-point bending forming process. The bending moment distribution during the forming process is shown in Figure 3 [6]. It can be observed that the bending moment distribution is more uniform and exceeds *M*_s_ compared to that of three-point bending process. Therefore, by improving the forming efficiency, the length of residual straight segment and the ellipticity of the formed pipe are reduced. Subsequently, H.J. [24] established a JCO forming CAPP system for LSAW pipe based on the SFB process and determined the formulation criteria of each process parameter. However, the influence of friction was ignored in this work, therefore the process parameters determined under this criterion will result in errors. Z.Z et al. [6] analyzed the SFB forming process while considering friction and other factors, reconstructed the mechanical model of the SFB process, and provided analytical expressions for the rotation angle and ordinate of any point during the forming process. With the increase in pass width, the deformation of the bending center is smaller, to the extent that plastic deformation may be absent.

In summary, while the SFB process addresses the issues of excessive forming passes and low efficiency in the JCO forming process of LSAW pipes to a certain extent, and contributes to a more uniform curvature distribution in the formed part, residual straight segments remain near the die contact point. Additionally, the deformation of the middle section fails to meet expectations, which inevitably impacts pipe quality and presents challenges during the final diameter expansion and roundness correction stages.

To further reduce residual straight segments and ellipticity, thereby improving forming accuracy, this paper proposes an asymmetric four-point air bending (AFB) process for the JCO forming process and establishes a corresponding mechanical model. In this context, asymmetry refers to the differing contact conditions between the left and right sides of the sheet. Subsequently, the AFB process and its bending moment distribution are analyzed.

**Figure 4:**
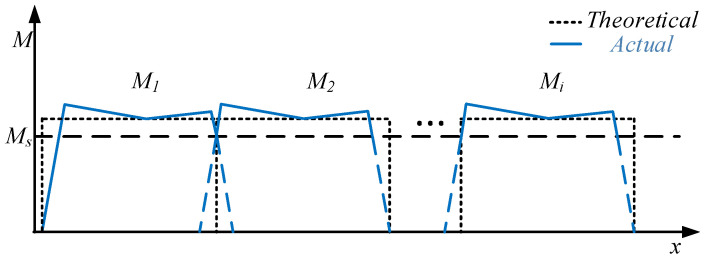
Bending moment distribution of JCO forming with the AFB process.

The bending moment distribution of the AFB process is shown in Figure 4. It can be observed that although the bending moment of the middle section gradually decreases due to friction, it remains relatively uniform and exceeds *M*_s_. Consequently, compared with the SFB process, the AFB process can further reduce the residual straight segment and the ellipticity of the formed pipe, thereby facilitating subsequent welding and rounding processes.

## 2. Establishment and Analysis of the Mechanical Model for the Asymmetrical Four-Point Air Bending Process

### 2.1. Basic Assumptions

**Figure 5:**
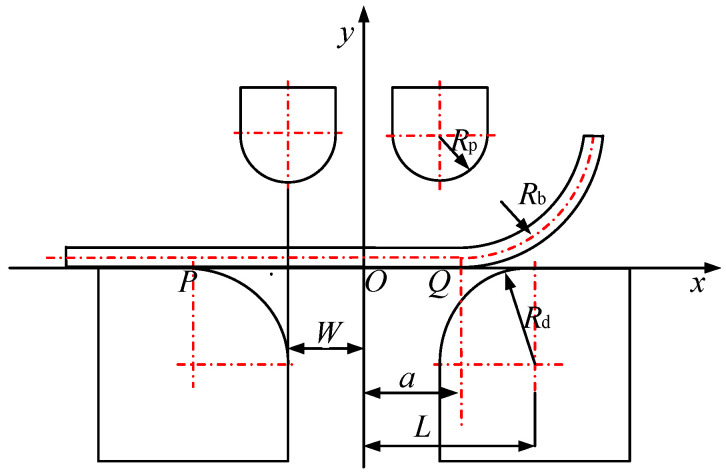
Mechanical model of asymmetrical four-point air bending.

As the JCO forming of LSAW pipe with the AFB process is essentially a multipass bending incremental process, a single pass is therefore selected as the subject of study. The initial state of the AFB process is shown in Figure 5.

The mold is symmetrically distributed on both sides, with the sheet positioned such that one end as a straight segment on the upper surface of the left die, while the other end is assumed to be a uniformly curved circular arc suspended above the right die. $R_{b}$ is the curvature radius of the arc segment. a is the distance from the symmetry axis to the transition point between the straight segment and the arc segment. $R_{p}$ is the radius of the punch. $R_{d}$ is the radius of the die. W is the distance from the center of punch to the symmetry axis. L is the distance from the center of die to the symmetry axis. Point P is the contact point between the sheet and the left die. Point Q is the transition point between the straight segment and the arc segment.

To facilitate the analysis of the AFB mechanical model, a planar coordinate system is established with the plane on the die as the *X*-axis and the symmetry axis as the *Y*-axis. Additionally, the basic rules of sheet deformation behavior are hypothesized.

1. Pure bending assumption: The bending process is considered as a pure bending process, meaning that the influence of normal stress is ignored and only bending strain is taken into account;
2. Neutral layer coinciding assumption: During the deformation process, the neutral layer of strain, the neutral layer of stress, and the geometric neutral layer always coincide;
3. The bilinear hardening material model assumption: Within the range of small plastic deformation, the hardening curve can be approximated by a straight line. The relationship between strain and stress is
  (1) $$\sigma =\left\{\begin{array}{l} \begin{array}{l} E\varepsilon \;\left(\varepsilon \leq \sigma_{s}/E\right) \end{array}\\ \begin{array}{l} D\varepsilon +\sigma_{0}\;\left(\varepsilon >\sigma_{s}/E\right) \end{array} \end{array}\right.$$
  (2) $$\sigma_{0}=\left(1-D/E\right)\sigma_{s}$$
  where *E* is Young’s modulus, σ_s_ is yield stress, σ_0_ is intercept stress, and *D* is plastic tangent modulus.

4. Planar section assumption: Any plane section remains a plane after deformation with no aberrance occurring. Therefore, the strain distribution across any section is linear, and the strain can be expressed as
  (3) $$\varepsilon =\frac{\omega }{\rho }$$
  where *ε* is strain, *ω* is the distance from the neutral layer, and *ρ* is the curvature of neutral layer after loading.

5. Uniaxial stress state assumption: Any particle of the sheet is uniaxially stretched or compressed when deformation occurs.

### 2.2. Bending Process and Springback Analysis

**Figure 6:**
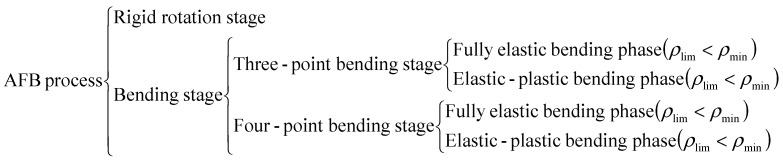
Stage division of AFB process. Where $\rho_{\lim}$ is the curvature radius when the sheet reaches the elastic limit state.

Due to the continuous changes in the force state of the sheet, the AFB process can be divided into distinct stages, as shown in Figure 6.

#### 2.2.1. Rigid Rotation Stage

**Figure 7:**
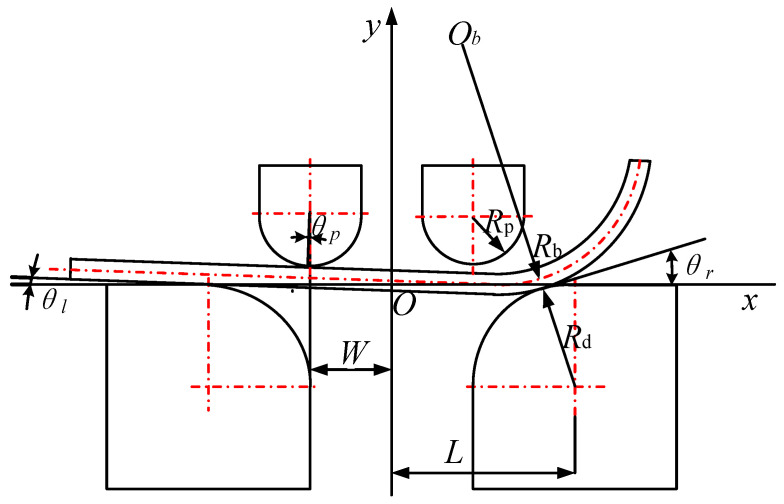
Mechanical model of rigid rotation stage.

During the rigid rotation stage, as shown in Figure 7, the punch moves downward, causing the sheet to rotate around the left die until the arc section comes into contact with the right die.

The contact point $P\left(x,y\right)$ between the sheet and the left die moves in a cycloidal path during the rigid rotation stage and reaches point $P^{\prime }\left(x^{\prime },y^{\prime }\right)$. According to the cycloidal equation,

(4) $$y^{\prime }=R_{d}\left(\cos\theta_{l}+\theta_{l}\sin\theta_{l}\right)-R_{d}$$

(5) $$x^{\prime }=R_{d}\left(\sin\theta_{l}-\theta_{l}\cos\theta_{l}\right)-L$$

where $\theta_{l}$ is the rotational angle on the contact point $P\left(x,y\right)$.

Meanwhile, point $Q\left(a,0\right)$ moved to point $Q^{\prime }\left(x_{q}^{\prime },y_{q}^{\prime }\right)$,

(6) $$\left\{\begin{array}{l} y_{q}^{\prime }=y^{\prime }-\left(L+a\right)\sin\theta_{l}\\ x_{q}^{\prime }=x^{\prime }+\left(L+a\right)\cos\theta_{l} \end{array}\right.$$

At this moment, point $Q\left(a,R_{b}\right)$, which is the center of the arc segment, moves to point $O_{b}^{\prime }\left(x_{b}^{\prime },y_{b}^{\prime }\right)$,

(7) $$\left\{\begin{array}{l} {y_{b}}^{\prime }={y_{q}}^{\prime }+\left(R_{b}+t/2\right)\cos\theta_{l}\\ {x_{b}}^{\prime }={x_{q}}^{\prime }+\left(R_{b}+t/2\right)\sin\theta_{l} \end{array}\right.$$

According to Equations (4)–(7),

(8) $$\left\{\begin{array}{l} {y_{b}}^{\prime }=\left(R_{d}\theta_{l}-L-a\right)\sin\theta_{l}+\left(R_{d}+R_{b}+t/2\right)\cos\theta_{l}-R_{d}\\ {x_{b}}^{\prime }=\left(R_{d}+R_{b}+t/2\right)\sin\theta_{l}\left(L+a-R_{d}\theta_{l}\right)\cos\theta_{l}-L \end{array}\right.$$

Since the distance from the point $O_{b}^{\prime }\left(x_{b}^{\prime },y_{b}^{\prime }\right)$ to the center of the right die is $\left(R_{b}+t/2\right)+R_{d}$, an equation can be derived:

(9) $${\left({x_{b}}^{\prime }-L\right)}^{2}+{\left({y_{b}}^{\prime }+R_{d}\right)}^{2}={\left(R_{b}+t/2+R_{d}\right)}^{2}$$

The point $O_{b}^{\prime }\left(x_{b}^{\prime },y_{b}^{\prime }\right)$ and $\theta_{l}$ can be obtained from Equations (8) and (9), and then, the $\theta_{r}$, which is the angle between right common tangent and *X*-axis, can be obtained too.

During the rigid rotation stage, the punch only needs to overcome the gravitational force of the sheet. Given that the calculation is more complex and the required pressure is relatively small, this pressure is therefore ignored in the calculation process.

#### 2.2.2. Three-Point Bending Stage

**Figure 8:**
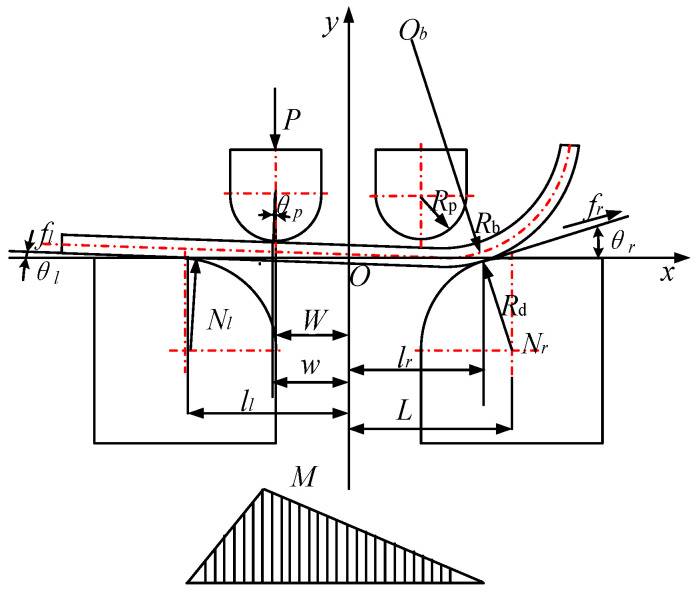
Mechanical model and moment distribution in three-point bending stage.

After the rigid rotation of the sheet is completed, the punch continues to move downward to enter the three-point bending stage, as shown in Figure 8.

At this stage, only the left punch is in contact with the sheet, the $\theta_{l}$ turn to $\theta_{l}^{\prime }$, the $\theta_{r}$ turn to $\theta_{r}^{\prime }$ and the $\theta_{p}$ turn to $\theta_{p}^{\prime }$. According to the statics analysis, it can be determined that

(10) $${\theta_{p}}^{\prime }=\theta_{p}+\left({\theta_{l}}^{\prime }-\theta_{l}\right)-\left({\theta_{r}}^{\prime }-\theta_{r}\right)$$

(11) $$l_{l}=L-\left(R_{d}+\frac{t}{2}\right)\sin{\theta_{l}}^{\prime }$$

(12) $$l_{r}=L-\left(R_{d}+\frac{t}{2}\right)\sin{\theta_{r}}^{\prime }$$

(13) $$w=W+\left(R_{p}+\frac{t}{2}\right)\sin{\theta_{p}}^{\prime }$$

(14) $$N_{l}=\frac{P\sin{\theta_{r}}^{\prime }}{\cos{\theta_{p}}^{\prime }\left(\sin{\theta_{r}}^{\prime }\cos{\theta_{l}}^{\prime }+\cos{\theta_{r}}^{\prime }\sin{\theta_{l}}^{\prime }\right)}$$

(15) $$N_{r}=\frac{P\sin{\theta_{l}}^{\prime }}{\cos{\theta_{p}}^{\prime }\left(\sin{\theta_{r}}^{\prime }\cos{\theta_{l}}^{\prime }+\cos{\theta_{r}}^{\prime }\sin{\theta_{l}}^{\prime }\right)}$$

(16) $$f_{l}=\mu N_{l}$$

(17) $$f_{r}=\mu N_{r}$$

where μ is coefficient of friction, $\theta_{l}$ is the rotation angle at the contact point between the sheet and the left die, $\theta_{r}$ is the rotation angle at the contact point between the sheet and the right die, $\theta_{p}$ is the rotation angle at the contact point between the sheet and the left punch, P is the forming force, $N_{l}$ is the left die contact point reaction force, $N_{r}$ is the right die contact point reaction force, t is the sheet thickness, w is the distance from the left punch contact point to the symmetry axis, $l_{l}$ is the distance from the left die contact point to the symmetry axis, and $l_{r}$ is the distance from the right die contact point to the symmetry axis.

The bending moment at a point on the neutral layer is given by $M_{x}$,

(18) $$M_{x}=\left\{\begin{array}{l} \begin{array}{ll} N_{l}\left(l_{l}+x\right)\frac{1}{\cos{\theta_{l}}^{\prime }} & \left(x\in \left[-l_{l},-w\right]\right) \end{array}\\ \begin{array}{ll} N_{r}\left(l_{r}-x\right)\frac{1}{\cos{\theta_{r}}^{\prime }} & \left(x\in \left(-w,l_{r}\right]\right) \end{array} \end{array}\right.$$

The three-point bending stage can be divided into the fully elastic bending phase and the elastic-plastic bending phase base on whether the curvature radius of a particle within the sheet is less than $\rho_{\lim}$.

1. Fully elastic bending phase

At this stage, the bending radius at any point of the sheet neutral layer satisfies the equation:

(19) $$\frac{1}{\rho_{x}}=\frac{M_{x}}{EI}$$

where $\rho_{x}$ is bending radius at any point of the sheet neutral layer.

At this time, the form is identical to that of the fully elastic bending stage in the SFB process [6], and it can be determined that

(20) $$\sin\theta_{x}=\int \frac{M_{x}}{EI}dx$$

With the boundary condition $\theta_{x}{|}_{x=-w}=\theta_{p}$, it can be derived that

(21) $$\theta_{x}=\left\{\begin{array}{l} \begin{array}{ll} \arcsin\int_{x}^{-w}\frac{M_{x}}{EI}dx+\theta_{p} & \left(x\in \left[-l_{l},-w\right]\right) \end{array}\\ \begin{array}{ll} \left|\arcsin\int_{-w}^{x}\frac{M_{x}}{EI}dx-\theta_{p}\right| & \left(x\in \left(-w,l_{r}\right]\right) \end{array} \end{array}\right.$$

Meanwhile, based on the geometric relationship, it can be derived that

(22) $$y{|}_{x=-l_{l}}=\left(R_{d}+\frac{t}{2}\right)\left(\cos{\theta_{l}}^{\prime }-1\right)$$

(23) $$y{|}_{x=l_{r}}=\left(R_{d}+\frac{t}{2}\right)\left(\cos{\theta_{r}}^{\prime }-1\right)$$

It can therefore be known that

(24) $$y=\left\{\begin{array}{l} \left(R_{d}+\frac{t}{2}\right)\left(\cos{\theta_{l}}^{\prime }-1\right)-\int_{-l_{l}}^{x}\tan\theta_{x}dx\;\left(x\in \left[-l_{l},-w\right]\right)\\ \left(R_{d}+\frac{t}{2}\right)\left(\cos{\theta_{r}}^{\prime }-1\right)-\int_{x}^{l_{r}}\left(\arcsin\int_{-w}^{x}\frac{M_{x}}{EI}dx-\theta_{p}\right)dx\;\left(x\in \left(-w,l_{r}\right]\right) \end{array}\right.$$

At this time, parameter h, which represents the reduction in the punch, can be calculated based on geometric relationships.

(25) $$h=R_{p}-\left(R_{d}+\frac{t}{2}\right)\left(\cos{\theta_{l}}^{\prime }-1\right)+\int_{-l_{l}}^{x}\tan\theta_{x}dx-\left(R_{p}+\frac{t}{2}\right)\cos\theta_{p}$$

If unloading is performed at this phase, the sheet will fully return to its original state due to its elastic bending condition. At the point where the longitudinal coordinate is x=W before the springback, if the y-coordinate is always less than −h−t/2, the sheet will enter the elastic-plastic bending phase; otherwise, it will enter the four-point bending stage.

2. Elastic-plastic bending phase

At this phase, the sheet adjacent to the left punch transitions into the elastic-plastic bending phase, while the right punch remains disengaged from the sheet. Concurrently, the elastoplastic boundary on the left side is located at point $P_{s1}\left(x_{s1},y_{s1}\right)$, and that on the right side at point $P_{s2}\left(x_{s2},y_{s2}\right)$, with the bending moments at both points correspond to the elastic limit moment,

(26) $$M_{\lim}=\frac{EI}{\rho_{\lim}}=\frac{EI}{\left(\frac{Et}{2\sigma_{s}}\right)}=\frac{2\sigma_{s}I}{t}$$

When $x\in \left[x_{s1},x_{s2}\right]$, there is

(27) $$M_{x}\geq M_{\lim}$$

Substituting Equation (26) into Equation (18) enables the calculation of $x_{s1},x_{s2}$. Consequently, $x\in \left[-l_{l},x_{s1}\right)$ and $x\in \left(x_{s2},l_{r}\right]$ represent the elastic deformation region, whereas $x\in \left[x_{s1},x_{s2}\right]$ represents the elastic-plastic deformation region, b represents the width of the sheet, A represents the cross-sectional area of the sheet at the x, ν represents the distance from this point to the neutral layer of the sheet. The bending moment within this latter region conforms to

(28) $$\begin{array}{ll} M_{x} & =\int \sigma \nu dA=2\int_{0}^{a}bE\varepsilon \nu d\nu +2\int_{a}^{\frac{t}{2}}b\left(\sigma_{0}+D\varepsilon \right)\nu d\nu \\ & =\left(\frac{2Eb\varepsilon_{s}^{3}}{3}-\sigma_{0}b\varepsilon_{s}^{2}-\frac{2Db\varepsilon_{s}^{3}}{3}\right)\rho_{x}^{2}+\frac{\sigma_{0}bt^{2}}{4}+\frac{Dbt^{3}}{12\rho_{x}} \end{array}$$

By substituting Equation (18), the bending curvature of the plastic deformation region can be calculated by the following two equations.

(29) $$\left\{\begin{array}{l} \begin{array}{cc} \psi_{l}\left(x,P\right)=\frac{1}{\rho_{x}} & \left(x\in \left[x_{s1},-w\right]\right) \end{array}\\ \begin{array}{cc} \psi_{r}\left(x,P\right)=\frac{1}{\rho_{x}} & \left(x\in \left(-w,x_{s2}\right]\right) \end{array} \end{array}\right.\;(\mathrm{unit}:\;1/\mathrm{mm})$$

By combining the boundary condition $\theta_{x}{|}_{x=-w}=\theta_{p}$ with Equation (21), the rotation angle at any point of the elastic-plastic region can be calculated.

(30) $$\theta_{x}=\left\{\begin{array}{l} \begin{array}{cc} \arcsin\int_{x}^{-w}\psi_{l}\left(x,P\right)dx+\theta_{p} & \left(x\in \left[x_{s1},-w\right]\right) \end{array}\\ \begin{array}{cc} \left|\arcsin\int_{x}^{-w}\psi_{r}\left(x,P\right)dx-\theta_{p}\right| & \left(x\in \left(-w,x_{s2}\right]\right) \end{array} \end{array}\right.$$

By combining the boundary conditions $\theta_{x}{|}_{x=x_{s1}}=\theta_{s1}$, $\theta_{x}{|}_{x=x_{s2}}=\theta_{s2}$ and Equation (21), the rotation angle in the fully elastic bending region can be determined.

(31) $$\theta_{x}=\begin{array}{cc} \arcsin\int_{x}^{-x_{s1}}\frac{M_{x}}{EI}dx+\theta_{s1} & \left(x\in \left[-l_{l},x_{s1}\right]\right) \end{array}$$

(32) $$\theta_{x}=\begin{array}{cc} \left|\arcsin\int_{x}^{-x_{s1}}\frac{M_{x}}{EI}dx-\theta_{s2}\right| & \left(x\in \left[x_{s2},l_{r}\right]\right) \end{array}$$

The ordinate of the corresponding region can be obtained from Equations (22) and (23) and Equations (30)–(32).

(33) $$y=\left\{\begin{array}{l} \begin{array}{cc} \left(R_{d}+\frac{t}{2}\right)\left(\cos{\theta_{l}}^{\prime }-1\right)-\int_{-l_{l}}^{x}\tan\theta_{x}dx & \left(x\in \left[-l_{l},-w\right]\right) \end{array}\\ \begin{array}{cc} \left(R_{d}+\frac{t}{2}\right)\left(\cos{\theta_{r}}^{\prime }-1\right)-\int_{x}^{l_{r}}\tan\theta_{x}dx & \left(x\in \left(-w,l_{r}\right]\right) \end{array} \end{array}\right.$$

At this point, the reduction in punch h is

(34) $$h=R_{p}-\left(R_{d}+\frac{t}{2}\right)\left(\cos{\theta_{l}}^{\prime }-1\right)+\int_{-l_{l}}^{x}\tan\theta_{x}dx-\left(R_{p}+\frac{t}{2}\right)\cos\theta_{p}$$

If the unloading is performed at this phase, according to the springback equation of small curvature plane bending [25], the curvature radius $\rho_{x}^{u}$ of elastic-plastic deformation region $x\in \left(x_{s1},x_{s2}\right]$ after springback is

(35) $$\frac{1}{\rho_{x}^{u}}=\left\{\begin{array}{l} \begin{array}{cc} \frac{1}{\rho_{x}}-\frac{N_{l}\left(l_{l}+x\right)}{EI\cos{\theta_{l}}^{\prime }} & \left(x\in \left[x_{s1},-w\right]\right) \end{array}\\ \begin{array}{cc} \frac{1}{\rho_{x}}-\frac{N_{r}\left(l_{r}-x\right)}{EI\cos{\theta_{r}}^{\prime }} & \left(x\in \left(-w,x_{s2}\right]\right) \end{array} \end{array}\right.$$

By substituting Equation (29),

(36) $$\frac{1}{\rho_{x}^{u}}=\left\{\begin{array}{l} \begin{array}{cc} \psi_{l}\left(x,P\right)-\frac{N_{l}\left(l_{l}+x\right)}{EI\cos{\theta_{l}}^{\prime }} & \left(x\in \left[x_{s1},-w\right]\right) \end{array}\\ \begin{array}{cc} \psi_{r}\left(x,P\right)-\frac{N_{r}\left(l_{r}-x\right)}{EI\cos{\theta_{r}}^{\prime }} & \left(x\in \left(-w,x_{s2}\right]\right) \end{array} \end{array}\right.$$

The rotation angle $\theta_{x}^{u}$ of any point after springback is

(37) $$\theta_{x}^{u}=\left\{\begin{array}{l} \begin{array}{cc} \arcsin\int_{x}^{-w}\left(\psi_{l}\left(x,P\right)-\frac{N_{l}\left(l_{l}+x\right)}{EI\cos{\theta_{l}}^{\prime }}\right)dx+\theta_{p} & \left(x\in \left[x_{s1},-w\right]\right) \end{array}\\ \begin{array}{cc} \left|\arcsin\int_{x}^{-w}\left(\psi_{r}\left(x,P\right)-\frac{N_{r}\left(l_{r}+x\right)}{EI\cos{\theta_{r}}^{\prime }}\right)dx-\theta_{p}\right| & \left(x\in \left(-w,x_{s2}\right]\right) \end{array} \end{array}\right.$$

If the y-coordinate at the specified position x=W is greater than −h−t/2 when the unloading is not performed, then the sheet enters the four-point bending stage.

#### 2.2.3. Four-Point Bending Stage

**Figure 9:**
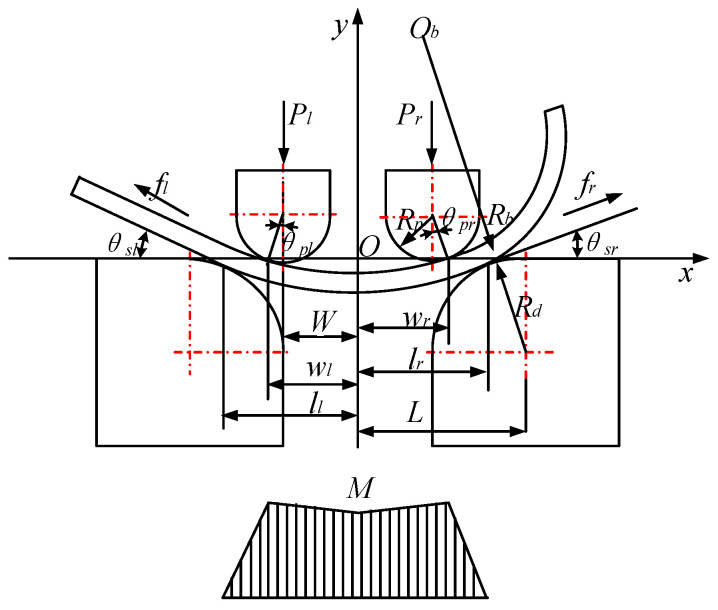
Mechanical model and moment distribution in the four-point bending stage.

As the right punch contacts the sheet, the four-point bending stage commences, resulting in changes to the mechanical model and bending moment, as shown in Figure 9, where $\theta_{sl}$ is the rotation angle at the contact point between the straight section of the sheet on the left side and *X*-axis, $\theta_{sr}$ is the rotation angle at the contact point between the circular arc section of the sheet on the right side and *X*-axis, $\theta_{pl}$ is the rotation angle between the line, which connects the contact point and center of the left punch, and *Y*-axis, $\theta_{pl}$ is the rotation angle between the line, which connecting the contact point and center of the right punch, and *Y*-axis. $P_{l}$ is the forming force of the left punch, $P_{r}$ is the forming force of the right punch, $w_{l}$ is the distance from the left punch contact point to the symmetry axis, and $w_{r}$ is the distance from the right punch contact point to the symmetry axis.

According to the statics analysis, it can be determined that

(38) $$l_{l}=L-\left(R_{d}+\frac{t}{2}\right)\sin\theta_{sl}$$

(39) $$l_{r}=L-\left(R_{d}+\frac{t}{2}\right)\sin\theta_{sr}$$

(40) $$w_{l}=W+\left(R_{p}+\frac{t}{2}\right)\sin\theta_{pl}$$

(41) $$w_{r}=W+\left(R_{p}+\frac{t}{2}\right)\sin\theta_{pr}$$

(42) $$P_{l}=\frac{P_{r}\sin\theta_{sr}\cos\theta_{pl}}{\sin\theta_{sl}\cos\theta_{pr}}$$

(43) $$N_{l}=\frac{P_{l}}{\cos\theta_{pl}}$$

(44) $$N_{r}=\frac{P_{r}}{\cos\theta_{pr}}$$

(45) $$f_{l}=\mu N_{l}$$

(46) $$f_{r}=\mu N_{r}$$

The bending moment $M_{x}$ of the sheet during the four-point bending stage is

(47) $$M_{x}=\left\{\begin{array}{l} \frac{N_{l}\left(l_{l}-x\right)}{\cos\theta_{sl}}-\mu N\left[\left(l_{l}+x\right)\sin\theta_{sl}+J_{l}\cos\theta_{sl}\right]\;\left(x\in \left(-l_{l},-w_{l}\right]\right)\\ \frac{N_{l}\left(l_{l}-w_{l}\right)}{\cos\theta_{sl}}-\mu N_{l}\left[\begin{array}{l} \left(l_{l}+w_{l}+2x\right)\sin\theta_{pl}+\\ \left(2J_{l}-\left(l-w\right)\tan\theta_{pl}\right)\cos\theta_{pl} \end{array}\right]\;\left(x\in \left(-w_{l},0\right]\right)\\ \frac{N_{r}\left(l_{r}-w_{r}\right)}{\cos\theta_{sr}}-\mu N_{l}\left[\begin{array}{l} \left(l_{r}+w_{r}-2x\right)\sin\theta_{pr}+\\ \left(2J_{r}-\left(l-w\right)\tan\theta_{pr}\right)\cos\theta_{pr} \end{array}\right]\;\left(x\in \left(0,w_{r}\right]\right)\\ \frac{N_{r}\left(l_{r}-x\right)}{\cos\theta_{sr}}-\mu N\left[\left(l_{r}-x\right)\sin\theta_{pr}+J_{r}\cos\theta_{pr}\right]\;\left(x\in \left(w_{r},l_{r}\right]\right) \end{array}\right.$$

(48) $$J_{l}=\left(R_{d}+\frac{t}{2}\right)\left(\cos\theta_{pl}-1\right)-y\;\begin{array}{c} x\in \left(-w_{l},0\right] \end{array}\;(\mathrm{unit}:\;\mathrm{mm})$$

(49) $$J_{r}=\left(R_{d}+\frac{t}{2}\right)\left(\cos\theta_{pr}-1\right)-y\;\begin{array}{c} x\in \left(0,w_{r}\right] \end{array}\;(\mathrm{unit}:\;\mathrm{mm})$$

(50) $$y=\left\{\begin{array}{l} \left(R_{d}+\frac{t}{2}\right)\left(\cos\theta_{sl}-1\right)-\left(l_{l}-w_{l}\right)\tan\theta_{sl}+\left(l_{l}+x\right)\tan\theta_{sl}\;\left(x\in \left(-l_{l},-w_{l}\right]\right)\\ \begin{array}{cc} \begin{array}{l} \left(R_{Tl}+R_{d}+\frac{t}{2}\right)\left(\cos\theta_{pl}-1\right)-\\ \left(l_{l}-w_{l}\right)\tan\theta_{pl}+R_{Tl}-\sqrt{R_{Tl}^{2}-x^{2}} \end{array} & \left(x\in \left(-w_{l},0\right]\right) \end{array}\\ \begin{array}{cc} \begin{array}{l} \left(R_{Tr}+R_{d}+\frac{t}{2}\right)\left(\cos\theta_{pr}-1\right)-\\ \left(l_{r}-w_{r}\right)\tan\theta_{pr}+R_{Tr}-\sqrt{R_{Tr}^{2}-x^{2}} \end{array} & \left(x\in \left(0,w_{r}\right]\right) \end{array}\\ \left(R_{d}+\frac{t}{2}\right)\left(\cos\theta_{pl}-1\right)-\left(l_{r}-w_{r}\right)\tan\theta_{pl}+\left(l-x\right)\tan\theta_{pl}\;\left(x\in \left(w_{r},l_{r}\right]\right) \end{array}\right.$$

(51) $$R_{Tl}=\frac{w_{l}}{\sin\theta_{pl}}+R_{p}+\frac{t}{2}\;\begin{array}{c} x\in \left(-w_{l},0\right] \end{array}\;(\mathrm{unit}:\;\mathrm{mm})$$

(52) $$R_{Tr}=\frac{w_{r}}{\sin\theta_{pr}}+R_{p}+\frac{t}{2}\;\begin{array}{c} x\in \left(0,w_{r}\right] \end{array}\;(\mathrm{unit}:\;\mathrm{mm})$$

When the three-point bending stage transitions to the four-point bending stage under fully elastic conditions, the four-point bending stage can be divided into the fully elastic bending phase and the elastic-plastic bending phase. Otherwise, only the elastic-plastic bending phase exists.

1. Fully elastic bending phase

At this phase, the state of the sheet is analogous to that of full elastic bending during the three-point bending stage. The bending radius can be calculated by Equation (19). Subsequently, the rotation angles at each point of the sheet can be obtained through the full elastic bending calculation method employed in the SFB process.

According to boundary conditions $\theta_{x}{|}_{x=-l_{l}}=\theta_{sl}$ and $\theta_{x}{|}_{x=l_{r}}=\theta_{sr}$, it is known that

(53) $$\theta_{x}=\left\{\begin{array}{l} \theta_{pl}-\arcsin\int_{-l_{l}}^{x}\frac{M_{x}}{EI}dx\;\left(x\in \left[-l_{l},-w_{l}\right]\right)\\ \theta_{-wl}-\arcsin\int_{-w_{l}}^{x}\frac{M_{x}}{EI}dx\;\left(x\in \left(-w_{l},0\right]\right)\\ \theta_{wr}-\arcsin\int_{x}^{w_{l}}\frac{M_{x}}{EI}dx\;\left(x\in \left(0,w_{r}\right]\right)\\ \theta_{pr}-\arcsin\int_{x}^{l_{r}}\frac{M_{x}}{EI}dx\;\left(x\in \left(w_{r},l_{r}\right]\right) \end{array}\right.$$

Meanwhile, based on the geometric relationship, it can be determined that

(54) $$y{|}_{x=-l_{l}}=\left(R_{d}+\frac{t}{2}\right)\left(\cos\theta_{sl}-1\right)$$

(55) $$y{|}_{x=l_{r}}=\left(R_{d}+\frac{t}{2}\right)\left(\cos\theta_{sr}-1\right)$$

The ordinate of the corresponding region can be obtained as

(56) $$y=\left\{\begin{array}{l} \left(R_{d}+\frac{t}{2}\right)\left(\cos\theta_{sl}-1\right)-\int_{-l_{l}}^{x}\tan\theta_{x}dx\;\left(x\in \left[-l_{l},-w_{l}\right]\right)\\ y_{-w_{l}}-\int_{-w_{l}}^{x}\tan\theta_{x}dx\;\left(x\in \left(-w_{l},0\right]\right)\\ y_{w_{r}}-\int_{x}^{w_{r}}\tan\theta_{x}dx\;\left(x\in \left(0,w_{r}\right]\right)\\ \left(R_{d}+\frac{t}{2}\right)\left(\cos\theta_{sr}-1\right)-\int_{x}^{l_{r}}\tan\theta_{x}dx\;\left(x\in \left(w_{r},l_{r}\right]\right) \end{array}\right.$$

At this point, the reduction in punch h is

(57) $$h=R_{p}-\left(R_{d}+\frac{t}{2}\right)\left(\cos\theta_{sl}-1\right)+\int_{-l_{l}}^{x}\tan\theta_{x}dx-\left(R_{p}+\frac{t}{2}\right)\cos\theta_{pl}$$

If unloading is performed at this phase, the sheet would fully return to its original state due to its elastic bending condition.

2. Elastic-plastic bending phase

At this phase, the sheet between the two punch contact points transitions into the elastic-plastic bending state. Concurrently, the elastoplastic boundary on the left side is located at point $P_{s1}\left(x_{s1},y_{s1}\right)$, and that on the right side at point $P_{s2}\left(x_{s2},y_{s2}\right)$. The bending moments at both points correspond to the elastic limit moment, which can be calculated by Equation (26).

Substituting Equation (26) into Equation (47) enables the calculation of $x_{s1},x_{s2}$. Consequently, $x\in \left[-l_{l},x_{s1}\right)$ and $x\in \left(x_{s2},l_{r}\right]$ denote the elastic deformation region, whereas $x\in \left[x_{s1},x_{s2}\right]$ represents the elastic-plastic deformation region.

By combining the boundary conditions $\theta_{x}{|}_{x=x_{s1}}=\theta_{s1}$, $\theta_{x}{|}_{x=l_{r}}=\theta_{sr}$ and Equation (21), the rotation angle in the fully elastic bending region can be obtained.

(58) $$\theta_{x}=\begin{array}{cc} \theta_{sl}-\arcsin\int_{l_{l}}^{x}\frac{M_{x}}{EI}dx & \left(x\in \left[-l_{l},x_{s1}\right]\right) \end{array}$$

(59) $$\theta_{x}=\begin{array}{cc} \theta_{sr}-\arcsin\int_{x}^{l_{r}}\frac{M_{x}}{EI}dx & \left(x\in \left[x_{s2},l_{r}\right]\right) \end{array}$$

Based on the distribution of bending moments in the four-point bending stage, the curvature at any point of the elastic-plastic region can be calculated:

(60) $$\left\{\begin{array}{l} \begin{array}{cc} \varphi_{l1}\left(x,P\right)=\frac{1}{\rho_{x}} & \left(x\in \left[x_{s1},-w_{l}\right]\right) \end{array}\\ \begin{array}{cc} \varphi_{l2}\left(x,P\right)=\frac{1}{\rho_{x}} & \left(x\in \left(-w_{l},0\right]\right) \end{array}\\ \begin{array}{cc} \varphi_{r2}\left(x,P\right)=\frac{1}{\rho_{x}} & \left(x\in \left(0,w_{r}\right]\right) \end{array}\\ \begin{array}{cc} \varphi_{r1}\left(x,P\right)=\frac{1}{\rho_{x}} & \left(x\in \left(w_{r},x_{s2}\right]\right) \end{array} \end{array}\right.\;(\mathrm{unit}:\;1/\mathrm{mm})$$

Combining boundary condition $\theta_{x}{|}_{x=x_{s1}}=\theta_{x_{s1}}$ and $\theta_{x}{|}_{x=x_{s2}}=\theta_{x_{s2}}$, the rotation angle at any point of the elastic-plastic region is as follows:

(61) $$\theta_{x}=\left\{\begin{array}{l} \theta_{s1}-\arcsin\int_{x_{s1}}^{x}\varphi_{l1}\left(P,x\right)dx\;\left(x\in \left[x_{s1},-w_{l}\right]\right)\\ \theta_{-w_{l}}-\arcsin\int_{-w_{l}}^{x}\varphi_{l2}\left(P,x\right)dx\;\left(x\in \left(-w_{l},0\right]\right)\\ \theta_{w_{r}}-\arcsin\int_{x}^{w_{r}}\varphi_{r2}\left(P,x\right)dx\;\left(x\in \left(0,w_{r}\right]\right)\\ \theta_{s2}-\arcsin\int_{x}^{x_{s2}}\varphi_{r1}\left(P,x\right)dx\;\left(x\in \left(w_{r},x_{s2}\right]\right) \end{array}\right.$$

The ordinate of the corresponding region can be obtained from Equations (54) and (55) and Equation (61).

(62) $$y=\left\{\begin{array}{l} \begin{array}{cc} \left(R_{d}+\frac{t}{2}\right)\left(\cos\theta_{sl}-1\right)-\int_{-l_{l}}^{x}\tan\theta_{x}dx & \left(x\in \left[-l_{l},0\right]\right) \end{array}\\ \begin{array}{cc} \left(R_{d}+\frac{t}{2}\right)\left(\cos\theta_{sr}-1\right)-\int_{x}^{l_{r}}\tan\theta_{x}dx & \left(x\in \left(0,l_{r}\right]\right) \end{array} \end{array}\right.$$

At this point, the reduction in punch h is

(63) $$h=R_{p}-\left(R_{d}+\frac{t}{2}\right)\left(\cos\theta_{sl}-1\right)+\int_{-l_{l}}^{x}\tan\theta_{x}dx-\left(R_{p}+\frac{t}{2}\right)\cos\theta_{pl}$$

If the unloading is performed at this phase, according to the springback equation of small curvature plane bending [25], the $\rho_{x}^{u}$ of elastic-plastic deformation region $x\in \left(x_{s1},x_{s2}\right]$ after springback is

(64) $$\frac{1}{\rho_{x}^{u}}=\left\{\begin{array}{l} \varphi_{l1}\left(x,P\right)-\frac{N_{l}\left(l_{l}-x\right)}{EI\cos\theta_{sl}}-\frac{\mu N\left[\left(l_{l}+x\right)\sin\theta_{sl}+J_{l}\cos\theta_{sl}\right]}{EI}\;\left(x\in \left(-l_{l},-w_{l}\right]\right)\\ \varphi_{l2}\left(x,P\right)-\frac{N_{l}\left(l_{l}-w_{l}\right)}{EI\cos\theta_{sl}}-\frac{\mu N_{l}\left[\begin{array}{l} \left(l_{l}+w_{l}+2x\right)\tan\theta_{pl}+\\ \left(2J_{l}-\left(l-w\right)\tan\theta_{pl}\right) \end{array}\right]}{EI\cos\theta_{pl}}\;\left(x\in \left(-w_{l},0\right]\right)\\ \varphi_{r2}\left(x,P\right)-\frac{N_{r}\left(l_{r}-w_{r}\right)}{EI\cos\theta_{sr}}-\frac{\mu N_{l}\left[\begin{array}{l} \left(l_{r}+w_{r}-2x\right)\tan\theta_{pr}+\\ \left(2J_{r}-\left(l-w\right)\tan\theta_{pr}\right) \end{array}\right]}{EI\cos\theta_{pr}}\;\left(x\in \left(0,w_{r}\right]\right)\\ \varphi_{r1}\left(x,P\right)-\frac{N_{r}\left(l_{r}-x\right)}{EI\cos\theta_{sr}}-\frac{\mu N\left[\left(l_{r}-x\right)\sin\theta_{pr}+J_{r}\cos\theta_{pr}\right]}{EI}\;\left(x\in \left(w_{r},l_{r}\right]\right) \end{array}\right.$$

The rotation angle $\theta_{x}^{u}$ of any point after springback is

(65) $$\theta_{x}^{u}=\left\{\begin{array}{l} \begin{array}{cc} \arcsin\int_{x}^{x_{s0}}\frac{1}{\rho_{x}^{u}}dx & \left(x\in \left[x_{s1},x_{s0}\right]\right) \end{array}\\ \begin{array}{cc} \arcsin\int_{x_{s0}}^{x}\frac{1}{\rho_{x}^{u}}dx & \left(x\in \left(x_{s0},x_{s2}\right]\right) \end{array} \end{array}\right.$$

By employing this equation in conjunction with Equation (62), the ordinate $y^{u}$ at any point after the springback can be determined.

(66) $$y^{u}=\left\{\begin{array}{l} \begin{array}{cc} -\int_{x_{s1}}^{x}\tan\theta_{x}^{u}dx+y^{u}{|}_{x=x_{s1}} & x\in \left[x_{s1},0\right] \end{array}\\ \begin{array}{cc} -\int_{x}^{x_{s2}}\tan\theta_{x}^{u}dx+y^{u}{|}_{x=x_{s2}} & x\in \left(0,x_{s2}\right] \end{array} \end{array}\right.$$

**Figure 10:**
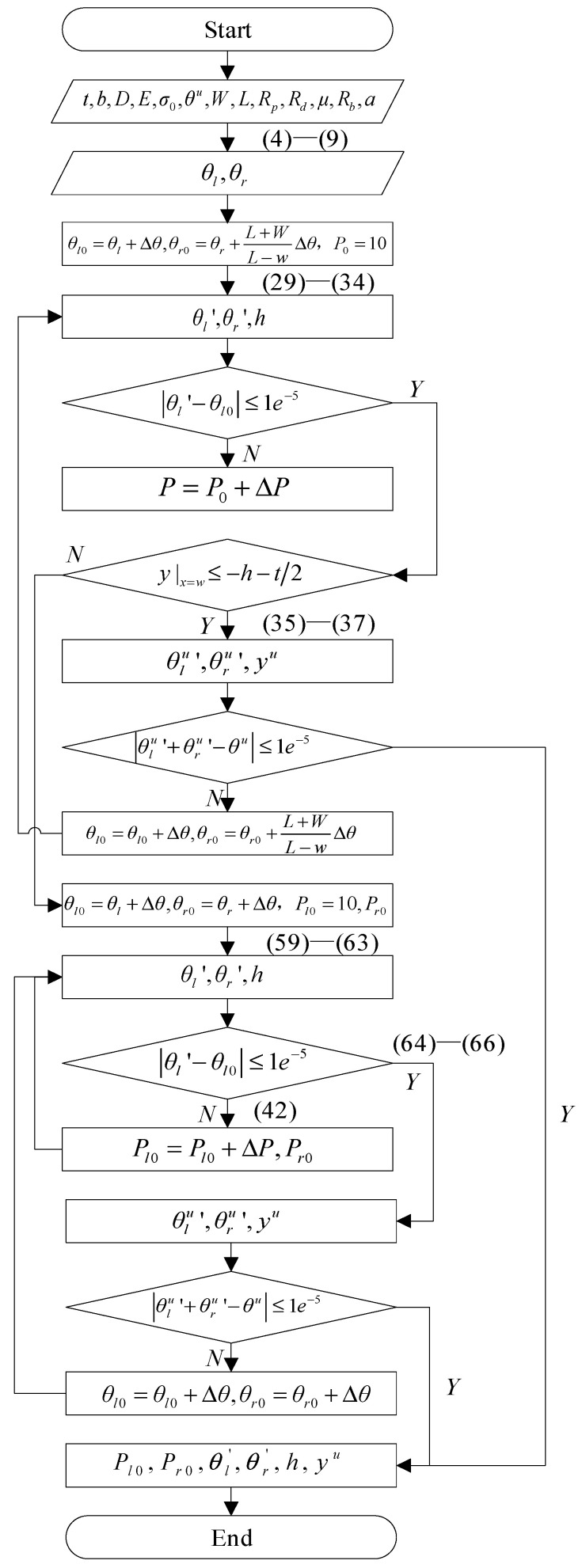
Flow chart of calculating the asymmetrical four-point bending mechanical model.

Based on the aforementioned theoretical model, the loading force P and the punch reduction h can be calculated, with the corresponding flow chart shown in Figure 10.

## 3. Finite Element Simulation and Experimental Verification

The AFB mechanical model and the corresponding JCO forming process were simulated and experimentally tested to verify their correctness.

**Figure 11:**
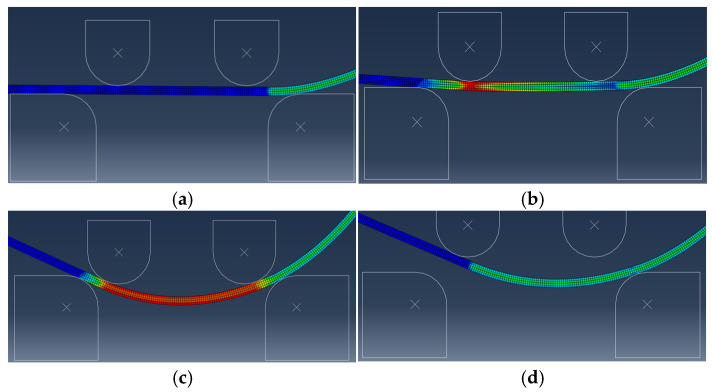
Simulation process of AFB mechanical model. (**a**) Rigid rotation stage; (**b**) Three-point bending stage; (**c**) Four-point bending stage; (**d**) Unload stage.

The finite element model is established by ABAQUS 6.14 software [26]. The sheet is discretized with 4-node bilinear plane strain quadrilateral elements featuring incompatible modes (CPE4I). The punches and dies are modeled as a discrete rigid body. The contact between the sheet and punch is set as a pure master–slave and kinematic contact condition with a frictional coefficient of 0.06. Additionally, the ABAQUS/Standard solver is employed. The simulation process of the AFB mechanical model is presented in Figure 11.

**Figure 12:**
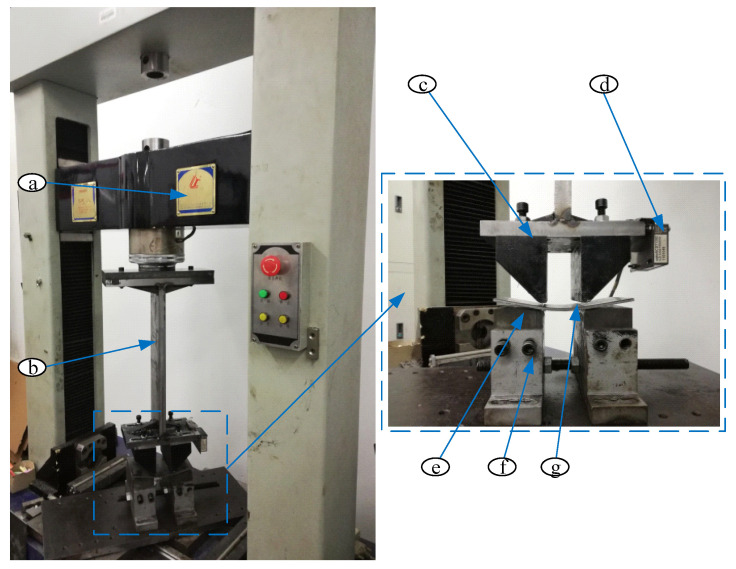
Experimental system. (**a**) test machine; (**b**) I-shape plate; (**c**) Punch; (**d**) Laser displacement sensor; (**e**) Die; (**f**) Slide; (**g**) Sheet.

The experimental system are developed according to the finite element model, including test machine, punches, dies, I-shape plate and laser displacement sensor, as shown in Figure 12. The punches are connected to the groove of the I-shaped plate, which is fixed on the crossbeam of the test machine, with adjustable spacing between them. The dies are mounted on two slides that are interconnected via screws, also featuring adjustable spacing between them.

The mechanical properties and geometric dimensions of the sheet, which are consistent with those of the finite element simulation, are presented in Table 1 and Table 2.

The process parameters of the AFB mechanical model are shown in Table 3, and *R*_b_ = 150 mm, 200 mm, 250 mm and *a* = 30 mm, 40 mm, 50 mm.

In industrial production, pipes with the same diameter-to-thickness ratio ρ/t are regarded as the same type, with this ratio ranging from 10 to 75. Considering the maximum forming force of the equipment and laboratory conditions, reduced proportion pipes with the ρ/t = 30, 37.5 and 50 were selected as the targets (the geometric dimensions of these pipes are shown in Table 4). The SFB and the AFB for JCO forming process were simulated and tested. The required process parameters are presented in Table 5 and Table 6.

**Figure 13:**
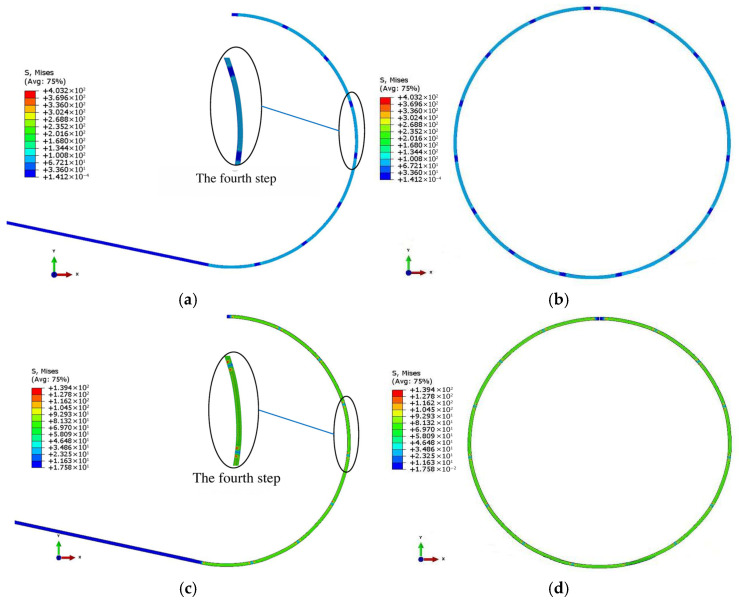
No. 2 pipe simulation results of SFB and AFB for JCO forming process. (**a**) Simulation results of SFB for JCO forming process; (**b**) overall simulation results of SFB for JCO forming process; (**c**) simulation results of AFB for JCO forming process; (**d**) overall simulation results of AFB for JCO forming process.

The No. 2 pipe simulation results of both SFB and AFB for the JCO forming process are shown in Figure 13.

**Figure 14:**
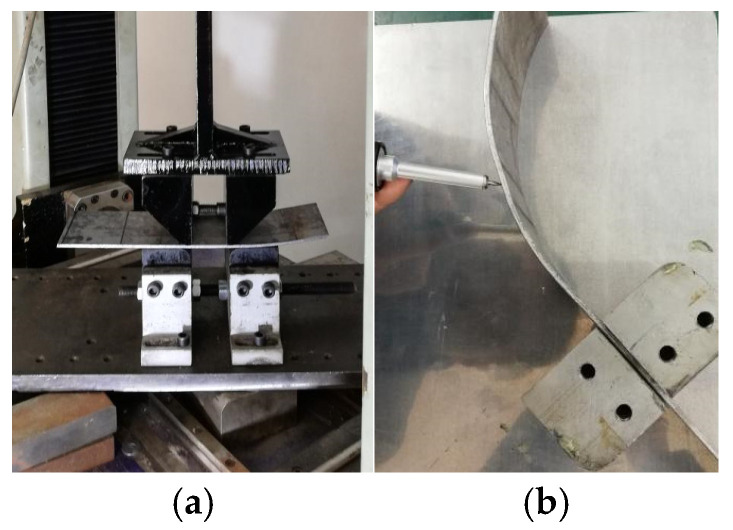
Experimental process and result of the AFB mechanical model. (**a**) Experimental process; (**b**) experimental results detection.

**Figure 15:**
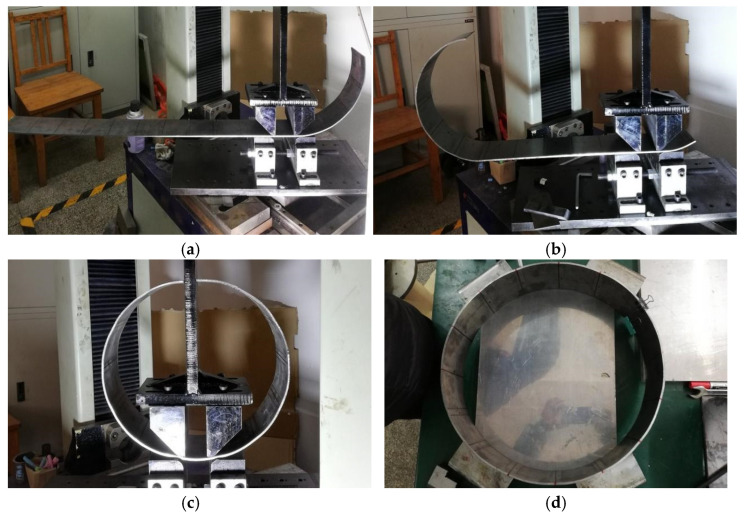
No. 2 pipe experimental process of AFB for JCO forming process. (**a**) J forming; (**b**) C forming; (**c**) O forming; (**d**) overall experimental results.

**Figure 16:**
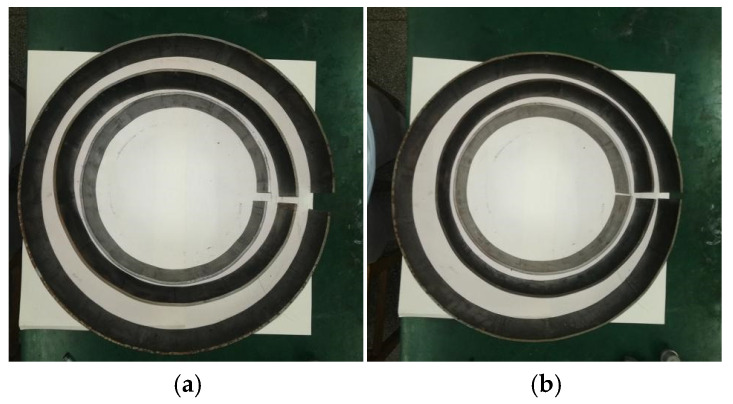
Three types of pipes experimental results of SFB and AFB for JCO forming process. (**a**) SFB process; (**b**) AFB process.

The experimental process of the AFB mechanical model are shown in Figure 14; the No. 2 pipe experimental process and results of AFB for JCO forming process are presented in Figure 15. Additionally, the three types of pipes experimental results of SFB and AFB for the JCO forming process are shown in Figure 16.

## 4. Simulation and Experimental Results Analysis

### 4.1. Results Analysis of AFB Mechanical Model

#### 4.1.1. Curvature Radius of the Arc Segment $R_{b}$ Analysis

Taking X80 sheet as the experimental material, the effects of rigid rotation, forming force and bending angle were investigated under conditions of a=30 mm and $R_{b}=150\;\mathrm{mm}$, $R_{b}=200\;\mathrm{mm}$ and $R_{b}=250\;\mathrm{mm}$, respectively.

**Figure 17:**
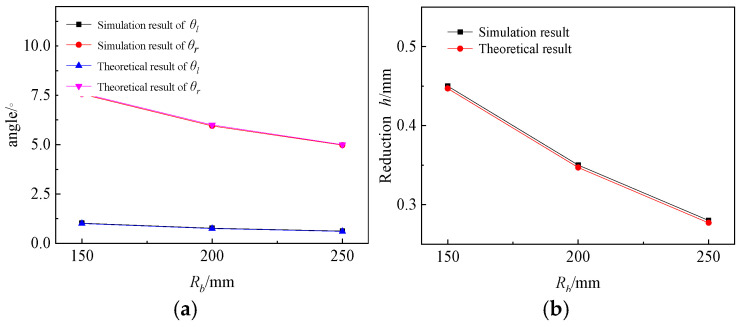
Angle and reduction after rigid rotation. (**a**) Angle result. (**b**) Reduction result.

1. Effects of rigid rotation:

As can be seen from Figure 17, the error between the simulation results and the theoretical analysis results is very small, which verifies that the rigid rotation stage is indeed that the sheet is expanded along the involute of the left die. The $\theta_{r}$ is always much larger than that of the $\theta_{l}$, and $\theta_{r}$ gradually decreases with the increase of $R_{b}$, with the decreasing range also decreasing as $R_{b}$ increases. Although $\theta_{l}$ follows this rule, its variation range is very small. This phenomenon is consistent with the situation where the AFB transitions to SFB when the $R_{b}$ is infinite. In general, the comparison of simulation results with theoretical analytical results verifies the reliability of rigid rotation calculations, which can quickly and effectively calculate the punch reduction h, $\theta_{l}$ and $\theta_{r}$ in the rigid rotation stage.

**Figure 18:**
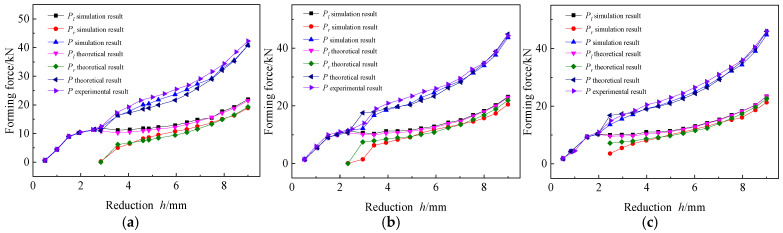
Effects of forming force. (**a**) $R_{b}=150\;\mathrm{mm}$; (**b**) $R_{b}=200\;\mathrm{mm}$; (**c**) $R_{b}=250\;\mathrm{mm}$.

2. Effects of forming force:

The forming force is an important parameter which plays a key role in the forming results of the established AFB mechanical model. Due to the difference in contact between the dies and the sheet on both sides during the forming process, the forming force changes in the left and right dies are studied respectively. However, the forming force cannot be measured individually during the experiment; therefore, only the total forming force is compared, as shown in Figure 18.

Both simulation result and theoretical result indicate that after completing rigid rotation and with a small amount of reduction h, the sheet is in an asymmetric three-point bending stage under the $P_{l}$, which increases linearly with the increase of h during the fully elastic bending phase. Although the $P_{l}$ increases linearly in the elastic-plastic bending state, its growth rate decreases. After the $P_{r}$ appears, the sheet transitions into the elastic-plastic bending phase of four-point bending stage. During the phase, as the h increases, the $P_{l}$ remains unchanged or increases only slightly, and then its growth rate continues to increase. Meanwhile, The $P_{r}$ increases rapidly after its appearance, and then continues to approach the $P_{l}$, although the $P_{l}$ is always greater than $P_{r}$. Both of them increases with the increase in the $R_{b}$ under the same h. However, the theoretical results of $P_{l}$ and $P_{r}$ underwent a sudden change when entering the four-point bending stage, and their trend of change became similar to the simulation and experimental results with the increase of h. In addition, the relative error between the theoretical result and the experimental result of P also decreased with the increase of h, verifying the correctness of the AFB mechanical model.

**Figure 19:**
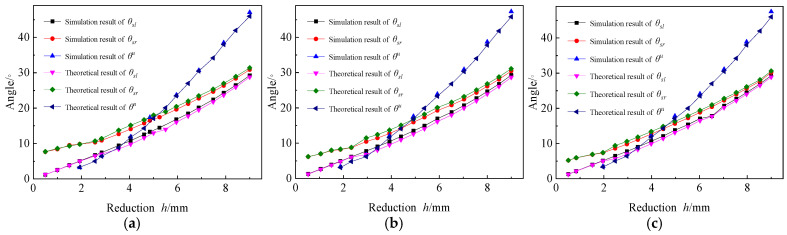
Effects of forming angle. (**a**) $R_{b}=150\;\mathrm{mm}$; (**b**) $R_{b}=200\;\mathrm{mm}$; (**c**) $R_{b}=250\;\mathrm{mm}$.

3. Effects of forming angle;

The forming angle is an important indicator for evaluating the forming results. Figure 19 presents the forming angles of different $R_{b}$ before and after springback under various h.

The simulation results of $\theta^{u}$, which is total forming angle after springback, show no significant difference from the theoretical results, both increase continuously with the increase of h, and the growth rate also increases accordingly. The simulation results during the three-point bending stage are basically the same as the theoretical results, and both $\theta_{sl}$ and $\theta_{sr}$ increase linearly. In the four-point bending stage, the simulation results of $\theta_{sl}$ is greater than the theoretical results, and the error of them is stable. The simulation results of $\theta_{sr}$ is smaller than the theoretical results, and the error is also stable. $\theta_{sl}$ is always lower than $\theta_{sr}$, but the error of them gradually decreases as the h increases.

The simulation results and theoretical results for all forming angles decrease continuously as the value of $R_{b}$ increase, However, the difference between these results remains stable, ranging between 0.2° and 0.4°, which indicates that $R_{b}$ has a minimal effect on the forming angle.

#### 4.1.2. Parameter a Analysis

To analyze the effect of the parameter a, defined as the distance from the symmetry axis to the transition point between the straight segment and the arc segment, on rigid rotation, bending force, and the angle, the AFB mechanical model was studied under the conditions of a=30 mm, a=40 mm, and a=50 mm, $R_{b}=150\;\mathrm{mm}$, with other parameters remaining the same as in the previous section.

**Figure 20:**
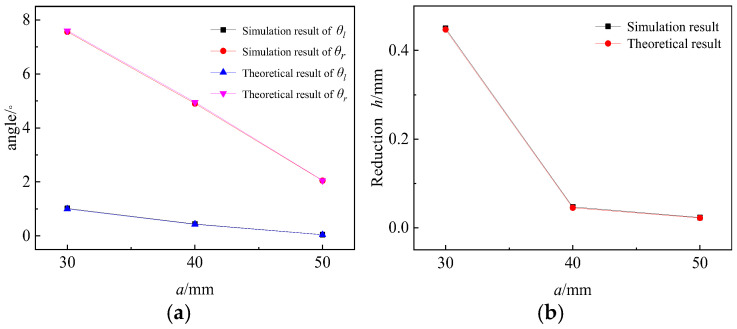
Angle and reduction after rigid rotation. (**a**) Angle result. (**b**) Reduction result.

1. Effects of rigid rotation;

As observed in Figure 20, there is minimal difference between the simulation results and the theoretical results, thereby verifying the calculation method of rigid rotation. The $\theta_{r}$ consistently remains significantly larger than $\theta_{l}$, with both parameters decreasing linearly as a increases. Notably, the decrease rate of $\theta_{r}$ is greater than that of $\theta_{l}$. The reduction h during rigid rotation not only exhibits a small difference but also decreases with the increase of a. Consequently, these findings indicate that the parameter a has a significant impact on rigid rotation.

**Figure 21:**
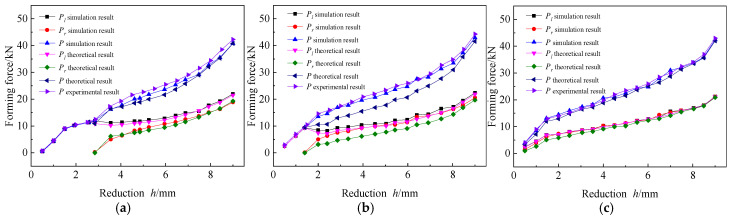
Effects of forming force. (**a**) a=30 mm; (**b**) a=40 mm; (**c**) a=50 mm.

2. Effects of forming force;

As shown in Figure 21, a=30 mm and a=40 mm, the sheet enter the elastic-plastic bending state first and then transitions to the four-point bending stage. In contrast, when a=50 mm, the sheet enters the four-point bending stage first and subsequently transitions to the elastic-plastic bending state. The $P_{l}$ decreases with the increase of a under the same h, while the $P_{r}$ increases with the increasing a, indicating that the difference between $P_{l}$ and $P_{r}$ gradually decreases as the a increase, bringing the results closer to those of the SFB process. Additionally, the error between the theoretical result and experimental result of P under different a decreases as the reduction h increases.

**Figure 22:**
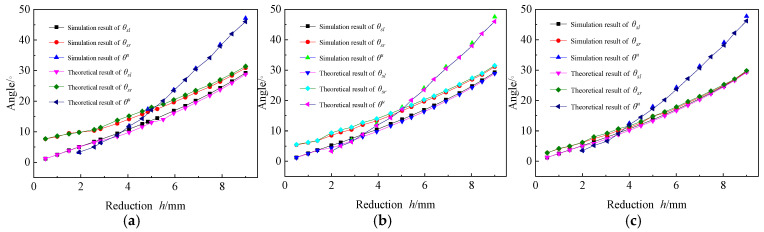
Effects of forming angle. (**a**) a=30 mm; (**b**) a=40 mm; (**c**) a=50 mm.

3. Effects of forming angle;

As illustrated in Figure 22, all forming angles increase with the increase in reduction h. The discrepancy between the simulation results and theoretical results decreases continuously with the increasing a, indicating that a larger a yields a more accurate AFB mechanical model. Throughout the bending process, $\theta_{sl}$ remains lower than $\theta_{sr}$, with the error between them gradually decreasing as both h and a increase. The difference of $\theta^{u}$ under varying a values are very small, suggesting that the larger a brings the AFB mechanical model closer to the SFB mechanical model. This parameter has a significant impact on $\theta_{sl}$ and $\theta_{sr}$, but exerts minimal influence on $\theta^{u}$.

### 4.2. Results Analysis of AFB for JCO Forming Process

As illustrated in Figure 14, pipes produced via the SFB forming process exhibit a short straight segment, uniform curvature distribution, and low ellipticity. In contrast, pipes manufactured using the AFB forming process demonstrate virtually no straight segment, with a curvature distribution that is more uniform and ellipticity that is smaller compared to those produced by the SFB forming process. This observation is consistent with the established AFB mechanical model.

The ellipticity of pipes can be calculated by

(67) $$\nabla =\frac{A_{\max}-A_{\max}}{D_{ia}}$$

where *A*_max_ is value of pipe major axis, *A*_min_ is the value of pipe minor axis, and *D_ia_* is the target diameter of pipe.

As shown in Table 7, the experimental forming results demonstrate that the ellipticity of pipes produced via the AFB process is similar and less than 0.66% (the minimum value is 0.53%), with simulation results for this process exhibiting ellipticity below 0.61% (the minimum value is 0.57%) and more stable compare to experimental results. The difference between experimental ellipticity and simulated ellipticity is greater than 0.02% and less than 0.08%. In contrast, the SFB process experimental results show an ellipticity of approximately 1%, while its simulation results indicate an ellipticity of 0.7%. The difference between experimental ellipticity and simulated ellipticity is greater than 0.23% and less than 0.34%. Compared to the SFB process, the ellipticity of pipes formed by the AFB process is reduced by approximately 33% and the difference between experimental ellipticity and simulated ellipticity are more consistent, indicating that correctness of the AFB mechanical model and the AFB process offers significant advantages in forming accuracy over the SFB process.

## 5. Conclusions

Aiming at the forming efficiency and roundness of the LASW pipes with the JCO forming process, this paper proposes a new forming process of asymmetric four-point bending (AFB) process. Furthermore, the verification experiments and simulations were conducted. The conclusions are as follows.

1. In the AFB process, the bending moment is evenly distributed between the punch points, resulting in an arc with uniform curvature for the LSAW pipes.
2. The AFB process can be divided into three stages based on the stress state: the rigid rotation stage, the asymmetric three-point bending stage, and the asymmetric four-point bending stage.
3. The forming force P, $P_{l}$, $P_{r}$, punch reduction h, forming angle $\theta^{u}$, $\theta_{sl}$, $\theta_{sr}$ and $y^{u}$ can be calculated based on the mechanical model of the AFB process.
4. The theoretical, simulation and experimental results of AFB mechanical model have verified the correctness of the established AFB mechanical model.
5. The effect of $R_{b}$ and a on rigid rotation, forming force, and forming angle was analyzed, providing experimental and theoretical foundations for the AFB JCO forming process of LASW pipes.
6. The simulation and experimental results of AFB for the JCO forming process demonstrate that the ellipticity of the pipes produced via the AFB process decreased by approximately 33% compared to that achieved through the SFB process. This indicates that the AFB process offers significant advantages in forming accuracy. These findings confirm the validity of the established AFB process, and the forming process has advantages over the SFB forming process, including high forming accuracy, fewer straight segments, and smaller ellipticity.

## Figures and Tables

**Table 1:** The mechanical properties of experimental sheet.

Material	Yield Strength *σ*_s_/MPa	Young’s Modulus *E*/MPa	Plastic Tangent Modulus *D*/MPa	Poisson’s Ratio ν
X80	450	197,000	7290	0.3
20 steel	282	210,000	2530	0.3

**Table 2:** Dimensions of sheet.

Material	Thickness /mm	Length /mm	Width /mm
X80	4	500	100
20 steel	5	500	100

**Table 3:** Process parameters of the AFB mechanical model.

Material	Thickness /mm	*R*_p_ /mm	*R*_d_ /mm	*W* /mm	*L* /mm	Width /mm	*μ*
X80	4	15	15	30	55	100	0.06

**Table 4:** Geometric dimensions of pipes.

Material	No.	Thickness /mm	Width /mm	ρ/t	ρ/mm
20 steel	1	5	100	30	150
	2			37.5	187.5
	3			50	250

**Table 5:** Process parameters of the SFB for JCO forming process.

No.	Sheet Length /mm	Passes *N*	Pass Length /mm	Forming Angle θ/°	*R*_p_ /mm	*R*_d_ /mm	*W* /mm	*L* /mm	Reduction h/mm	Forming Force P/kN
1	943	11	85	32.7	20	20	36	47.5	4.34	58.1
2	1178	15	78	24	15	15	27.5	42	2.86	66.5
3	1571	19	82	18.95	10	10	33	43	1.74	104.9

**Table 6:** Process parameters of the AFB for JCO forming process.

No.	Sheet Length /mm	Passes *N*	Pass Length /mm	Forming Angle θ/°	a /mm	*R*_p_ /mm	*R*_d_ /mm	*W* /mm	*L* /mm	Reduction h/mm	Forming Force P/kN
1	943	11	85	32.7	42.5	20	20	31	53	4.66	56.3
2	1178	15	78	24	39	15	15	30.5	45.5	2.88	67
3	1571	19	82	18.95	41	10	10	30.5	40.5	1.79	101

**Table 7:** Experiment and simulation forming dimensions of various pipes.

No.	Forming Process	Experimental Pipe Major Axis *A*_max_/mm	Experimental Pipe Minor Axis *A*_min_/mm	Experimental Pipe Ellipticity ∇/%	Simulation Pipe Major Axis *A*_max_/mm	Simulation Pipe Minor Axis *A*_min_/mm	Simulation Pipe Ellipticity ∇/%
1	SFB	306	303	0.98	306.3	304	0.75
	AFB	307	305	0.66	306.1	304.3	0.59
2	SFB	385	381	1.05	382.2	379.5	0.71
	AFB	384	382	0.53	381.9	379.6	0.61
3	SFB	509	504	0.99	507.6	504.1	0.69
	AFB	506	503	0.59	507.2	504.3	0.57

## Data Availability

The original contributions presented in this study are included in the article. Further inquiries can be directed to the corresponding author.

## Abbreviations

The following abbreviations are used in this manuscript:

LSAW	Large-scale longitudinally submerged arc welded
SFB	Symmetrical four-point bending
AFB	Asymmetrical four-point bending
CAPP	computer-aided process planning
